# A cost-utility analysis of cochlear implants for single sided deafness in adults and children in the Netherlands

**DOI:** 10.1371/journal.pone.0307881

**Published:** 2024-08-05

**Authors:** Chris van Lieshout, Katharina Abraham, Adriana L. Smit, Geert W. J. Frederix

**Affiliations:** 1 The Healthcare Innovation Center (THINC), Julius Center for Health Sciences and Primary Care, University Medical Center Utrecht, Utrecht University, Utrecht, The Netherlands; 2 Department of Epidemiology and Health Economics, Julius Center for Health Sciences and Primary Care, University Medical Center Utrecht, Utrecht University, Utrecht, The Netherlands; 3 Department of Otorhinolaryngology–Head and Neck Surgery, University Medical Center Utrecht, Utrecht University, Utrecht, The Netherlands; CNR, ITALY

## Abstract

**Background:**

Cochlear Implant (CI) has been shown to improve speech comprehension, sound localization and tinnitus in adults with Single-Sided-Deafness (SSD) compared to standard treatment currently available in the Dutch setting such as a CROS (Contralateral Routing of Signals) hearing device or a BCD (Bone Conduction Device). Also, for the pediatric population with SSD, CI has shown to be clinically meaningful. Because currently no information is available on the health economic effects of CI in adults and children with SSD in the Netherlands, a cost-utility analysis was conducted.

**Methods:**

We developed a Markov cohort model, for both the adult and pediatric SSD population, with three states: implant, no implant, and dead. CI was compared with the Bone Conduction Device (BCD) treatment, requiring surgery and no specific treatment. The time horizon of the model was lifelong, costs were discounted with 3% and effects with 1.5%. A societal perspective was taken, including productivity costs in the analysis, with costing data based on publicly available prices for the Netherlands. Values for clinical outcome parameters, i.e. hearing gain, and event probabilities were based on existing literature. Deterministic and probabilistic sensitivity analyses as well as scenario analyses were performed to outline uncertainty of individual and combined parameters.

**Results:**

Mean per patient costs for CI in the adult population were €194,051 (95%-CrI €177,274 to €211,108) compared to the total costs of €185,310 (95%-CrI €182,367 to €194,142) for BCD resulting in a cost difference of €8,826 (95%-CrI -€5,020 to €18,252). Compared to no treatment, the cost difference was -€25,089 (95%-CrI -€31,678 to -€6,003). Adults who were treated with CI gained 18.41 (95%-CrI 18.07 to 18.75) quality adjusted life years (QALY) whereas BCD patients gained 15.81 QALYs (95%-CrI 15.53 to 16.10), a difference of 2.60 QALYs (95%-CrI 2.15 to 3.05). The Incremental Cost Effectiveness Ratio (ICER) for adults with CI was determined to be €3,494/QALY gained. Patient without treatment gained 13.46 QALY (95%-CrI 13.20 to 13.73), a difference of 4.95 (95%-CrI 4.87 to 5.01) resulting in CI dominating no treatment. The ICER remained below the Dutch threshold of €20,000/QALY. The probabilistic sensitivity analyses confirmed the results. For children, CI dominated when compared to BCD and when compared to no treatment. Compared to BCD, CI led to a cost saving of €29,611 (95%-CrI -€126,800 to €54,375) and compared to no treatment, CI resulted in a cost saving of €57,658 (95%-CrI -€146,687 to €5,919). The incremental QALY gain compared to BCD was 7.22 (95%-CrI 4.19 to 8.55) and 26.03 (95%-CrI 20.82 to 31.06) compared to no treatment.

**Conclusions:**

Based on the results of this health economic evaluation with a Markov cohort model, it is very likely that CI is cost-effective compared to BCD and to no treatment in the Dutch adult and pediatric population with SSD. In both populations the ICER was below the Dutch cost-effectiveness threshold of €20,000/QALY.

## Introduction

Patients with single-sided deafness (SSD) have severe-to-profound hearing loss in one ear and normal or near-normal hearing in the other ear [1]. SSD can be present since birth or can be acquired over time due to several causes. SSD at birth is relatively uncommon in the Netherlands with 0.46 per 1,000 babies affected [2]. Disadvantages of hearing with only one ear relate to sound localization abilities and speech comprehension, especially in noisy environments [3]. This can result in fatigue, difficulties with keeping up in school, and difficulties with keeping social contacts. Lower rates of employment, early retirement and restricted career opportunities have also been associated with hearing impairment [4, 5]. For the Netherlands it was estimated that the employment rate is 10% lower in the population with hearing impairment resulting in an estimated €2 billion societal costs in 2013 [4].

Currently, SSD cannot be cured. Instead, available treatments aim at improving speech comprehension and sound localization by means of hearing devices. At present, in the Netherlands two treatment options are available for SSD: 1) CROS (Contralateral Routing of Signals) and 2) BCD (Bone Conduction Device) [6]. Before implantation of the BCD, patients are recommended to wear a headband (adults)/softband (children) on trial for a minimum of 14 days. An alternative to these devices might be the use of a CI (Cochlear Implant). A CI is an electronic device that electrically stimulates the cochlear nerve, which requires surgery to insert. This device is recommended in patients with severe bilateral deafness and limited speech perception with standard hearing devices to restore functional hearing. Based on outcomes of recent studies, in SSD cases, the hearing performance with regard to localization of sounds and speech comprehension improved although normal hearing is not restored [7–10]. Currently, CIs are not reimbursed in patients with SSD in most countries mainly due to the high costs associated with the implant [6]. Given the recent findings on improved speech comprehension, sound localization and the severity and occurrence of tinnitus, a Cost-Utility Analysis (CUA) was conducted for CI in children and adults with SSD in the Dutch setting to investigate whether reimbursement in these patients would be warranted.

## Methods

**Fig 1:**
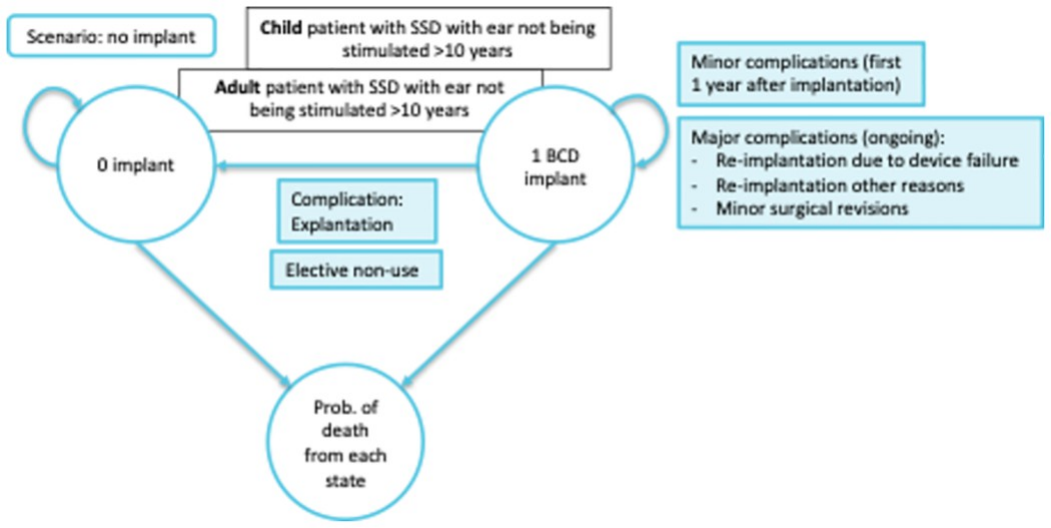
Schematic representation of the decision analytic model.

To calculate the expected health economic effects of CI compared with BCD and no treatment in patients with SSD, we developed a Markov decision analytical model (Fig 1). In a Markov model, a cohort of patients moves between defined health states, patients can be in one health state at a time. Patients move between states based on the probabilities found in scientific literature. The cycle length was 6 months, and the time horizon of the analysis was lifelong, meaning simulation ran until all patients were deceased.

The Markov model used in this analysis was based on a recent Health Technology Assessment (HTA) performed by Health Quality Ontario on CI for patients with SSD and consists of three mutually exclusive health states: 1) no implant, 2) implant, and 3) dead [11]. Having an implant is associated with a continued risk for major complications. Reimplantation is assumed to occur once and only in case of complications. Explantation of the implant and non-use of the device both result in the transitioning from the ‘implant’ state to the ‘no implant’ state in the model. In our model we assumed, once explanted the patient cannot receive a new implant and therefore remains in the ‘no implant’ state. Patients with and without implant have the same risk of death. In this Markov model, three cohorts of 1,000 patients are populated: One cohort receives BCD, one receives no specific treatment, and the other cohort receives CI as the new intervention. In a scenario analysis productivity costs were excluded and different cut-offs for ages were employed.

The analysis was performed from a limited societal perspective for the Dutch setting, thus in addition to direct healthcare costs, travel costs, and productivity costs were included in the analysis. Future medical costs and end-of-life costs were excluded as no difference in mortality is assumed for this analysis.

### Patient population and treatment

The patient population consists of adults (≥ 18 years) and children with SSD whose affected ear has not been deprived from auditory stimulation for more than 10 years. In this analysis CI was compared with BCD based on the fact that for both an invasive (surgical) procedure is needed. As children or adults with SSD commonly use no treatment or opt for no treatment given the perceived limited benefits with BCD, the no treatment group was added as a third group [12]. The health economic effects of CI vs no treatment is therefore also investigated. The mean age at model entry was 53 years (Range: 41 to 65) for the adult population and 1.5 years (Range 0.5 to 17) for the pediatric population (9, 11). 54% of the pediatric population and 48% of the adult population was assumed male [9, 11].

### Input parameters

For this health economic evaluation, we primarily used scientific literature to identify values for use in the model. When data was not available, literature-based assumptions were made.

### Clinical outcomes

Clinical outcomes for the pediatric and adult population were derived from two HTA reports by Health Quality Ontario [11, 13]. Major complications were categorized into device failure and non-device failure. Probabilities for device-/non-device failure for CI were calculated based on major complication probabilities and the distribution suggested by Wang et al. 2014 for device-/non-device failure [11, 13]. For BCD, the probability for device failure was derived from literature [11, 14]. In case of non-device failure there is a risk of explantation, re-implantation and minor revision. The probabilities for minor complications represent the risk of infections (of the skin, otitis media), neurological complications (e.g., facial palsy), pain, facial stimulation, tinnitus, vestibular complications and any other complications associated with the implantation and/or activation of hearing devices (e.g., cerebrospinal fluid leak hematoma) [13]. Studies stated a beneficial effect of CI on tinnitus compared to BCD in SSD patients [7, 9]. However, due to the lack of data on the incidence of tinnitus for BCD in these cases, we assumed the same risk as for CI in the base case analysis. In the sensitivity analysis the impact of this assumption on the cost-effectiveness is shown. Whereas most of the minor complications were assumed to occur within the first year after surgery, the probability of tinnitus occurring or worsening, the probability of vestibular complications and the probability of all major complications were assumed to continue across lifetime. The probability for elective non-use of the hearing devices in adult and pediatric patients with SSD was also derived from the Health Quality Ontario HTA report [11]. An overview of probabilities used in the model can be found in Table 1.

**Table 1:** Probabilities.

Adults	Mean	SE	Source
**Cochlear Implant**			
Device failure	0.0038	0.0004	[10, 11]
Explantation	0.0005	0.0001	[10, 11]
Re-implantation	0.0007	0.0001	[10, 11]
Other complications requiring revision	0.0019	0.0002	[10]
Minor complications	0.0690	0.0069	[11]
Tinnitus	0.0160	0.0016	[11]
Vestibular complications	0.0250	0.0025	[11]
Elective non-use	0.0095	0.0010	[10]
**Bone Conduction Device**			
Device failure	0.0014	0.0001	[12]
Explantation	0.0005	0.0001	[10]
Re-implantation	0.0004	0.0000	[10]
Other complications requiring revision	0.0020	0.0002	[10]
Minor Complications	0.0280	0.0028	[10]
Tinnitus	0.0160	0.0016	Assumption
Vestibular complications	0.0250	0.0025	Assumption
Elective Non-Use	0.0064	0.0006	[10]
**Children**	**Mean**	**SE**	**Source**
**Cochlear Implant**			
Device failure	0.0041	0.0004	[10, 11]
Explantation	0.0003	0.0000	[10, 11]
Re-implantation	0.0066	0.0007	[10, 11]
Other complications requiring revision	0.0024	0.0002	[10]
Minor complications	0.0330	0.0033	[11]
Tinnitus	0.0000	0.0000	[11]
Vestibular complications	0.0050	0.0005	[11]
Elective non-use	0.0026	0.0003	[10]
**Bone Conduction Device**			
Device failure	0.0014	0.0001	[12]
Explantation	0.0075	0.0008	[10]
Re-implantation	0.0052	0.0005	[10]
Other complications requiring revision	0.0204	0.0020	[10]
Minor Complications	0.5632	0.0563	[10]
Tinnitus	0.0000	0.0000	Assumption
Vestibular complications	0.0050	0.0005	Assumption
Elective Non-Use	0.0064	0.0006	[10]

### Costs

Table 2 shows the costs for all healthcare provided to SSD patients in the model. Costs are based on frequencies and unit costs. Costs were inflated to Euros 2023 based on consumer price indices provided by the Dutch Central Bureau for Statistics (CBS) [15, 16]. Where applicable travel costs were included. For most care consumption, we assumed the average distance to a hospital, according to the Dutch guideline [16, 17].

**Table 2:** Costs.

	Unit costs	SE	Source
**Pre-, post-, and procedural costs**			
Audiometry	€60	€6	[15]
Audiological assessment	€158	€16	[17]
Vestibular assessment	€158	€16	[17]
Consultation Speech therapist	€43	€4	[16]
Consultation Psychologist / Social worker	€97	€10	[16]
MRI	€131	€13	[15]
CT scan	€131	€13	[15]
Electronystagmography (ENG)	€258	€26	[15]
Surgical consult	€506	€51	[16]
Preoperative general assessment	€121	€12	[16]
CI + processor and surgery	€59,108	€5,911	Hopsital Prices
One-sided CI processor replacement	€14,533	€1,453	Hopsital Prices
BCD + processor and surgery	€9,334	€933	Hopsital Prices
Replacement BCD processor	€4,574	€457	Expert opinion
Physician	€173	€17	[16]
**Minor complication costs**			
Infection (skin, otitis media)	€145	€14	[16]
Neurological complications (facial palsy, dysgeusia)	€132	€13	[16]
Pain (facial stimulation, other)	€121	€12	[16]
Tinnitus (worsening or new occurrence)	€989	€99	[18]
Vestibular complications (vertigo, dizziness)	€238	€24	[16]
Other complications (cerebrospinal fluid leak hematoma, atlantoaxial subluxation)	€121	€12	[16]
**Major complication costs**			
Surgical costs CI (Explantation)	€3,652	€364	[15]
Surgical costs BCD (Explantation)	€398	€40	[15]
Re-implantation CI	€44,581	€4,458	Calculated
Re-implantation BCD	€4,765	€477	Calculated
Minor revision (infection, cholestaetoma, other)	€1,189	€119	Hospital Prices
**Productivity Costs**			
Hourly Productivity Costs Males	€48	€5	[16]
Hourly Productivity Costs Females	€40	€4	[16]

BCD: Bone Conudction Device, CI: Cochlear Implant, CT: Computed Tomography, MRI: Magnetic Resonance Imaging

Preprocedural and postprocedural healthcare consumption was derived from literature [11, 18]. Costing data for the healthcare resource use was mainly derived from the Nederlandse Zorgauthoriteit (Netherlands Healthcare Authority, NZa) and the Zorginstituut Nederland (The National Health Care Institute, ZIN) [16, 17]. For the costs of audiological and vestibular assessments, a study by Joore et al. was utilized [19]. Overall, CI resulted in more resource use compared to BCD prior to the surgery and in the first two years after the surgery but was less 2 years post-surgery [11, 18]. The procedural costs for BCD and CI including the implant, processor, and surgical costs were based on the average of the published prices 2021 from five University Medical Centers (UMCs). As part of standard healthcare, the processor of the CI and BCD is replaced every 5 years. The costs for the replacement of the processor for CI was based on the average of the UMC prices 2021. The replacement of the BCD processor was based on company data. Unit costs for major and minor complications were derived from the Dutch costing guideline, from Medicijkosten.nl and from literature [16, 17, 20, 21]. Reimplantation of the CI and BCD device was calculated as the difference in costs for device implantation and processor costs. Costs for other complications requiring revision were based on the average passenger tariffs from the five Dutch UMCs.

#### Productivity costs

For productivity costs we used the human capital approach estimating life-time losses due to presenteeism. The reason is that patients will benefit from treatment for their lifetime, whereas the absenteeism approach does not take benefits in the far future into account. To determine productivity we used labor data from Statistics Netherlands, including labor participation per age bracket and combined this with reference productivity values from the ZIN costing manual [16, 22]. To determine the productivity costs, we first determined the expected productivity for all working ages. Assuming a healthy utility of 0.9 we used proportionality of health state utilities to determine the productivity costs. For example, no implant has a utility of 0.56 (see Table 3) which is 62.2% of 0.90, thus productivity for a person without implant is 62.2% of the expected productivity for the age bracket the patient is in. Based on these assumptions we calculated the costs of being less productive due to hearing loss. For BCD productivity was 74.4% of expected productivity and for CI productivity was 88.9% compared to people without hearing loss.

**Table 3:** Health related quality of life utility.

Adults & Children	Mean	SE	Source
**HRQOL Utilities**			
No treatment	0.56	0.06	[7]
CI	0.80	0.08	[7]
BCD	0.67	0.07	[7]
**Complication HRQOL Disutilities**			
Minor complications	-0.02	-0.009	[10]
Major complications	-0.02	-0.0121	[10]
Tinnitus	-0.02	-0.0219	[11]
Vestibular complications	-0.03	-0.009	[11]

BCD: Bone Conduction Device, CI: Cochlear Implant, HRQOL: Health Related Quality of Life

### Health related quality of Life

In the analysis we used Quality Adjusted Life Years (QALY) to determine the effects of both treatments. In the model a health-related quality-of-life utility (HRQOL) is assigned to the health states (one implant/ no implant) and events (complications). HRQOL utilities for adults with SSD without implantation, BCD, and CI were derived from Arndt et al. [7]. In contrast to Dutch guidelines, the utilities were based on HUI-3 questionnaires. For the pediatric population we assumed the same HRQOL utilities as for adults due to the lack of data. For complications a utility decrement is used to reflect the temporary reduction in HRQOL [11, 13].

### Analysis of model outcomes

The main outcome of this study is the ICER (Incremental-Cost-Effectiveness ratio), a ratio between the differences in costs and effects, which reflects the additional costs for one year spent in perfect health (1 QALY).

CI is considered cost effective compared to BCD or no treatment if the ICER remains below the Dutch cost effectiveness threshold, which lies between € 20,000/QALY and € 80,000/QALY depending on the disease burden. In the case of adults with SSD the threshold lies at €20,000/QALY [23].

In line with the Dutch guidelines, costs were discounted with 3% and effects with 1.5%. Discounting is used to account for the time factor in future costs and effects [24]. To address the uncertainty surrounding parameter assumptions, we performed sensitivity analyses. The one-way sensitivity analysis (OWSA) was performed, varying individual parameters values with plus or minus 20%, to determine which of the parameters had the largest impact on cost effectiveness. A Probabilistic Sensitivity Analysis (PSA) was performed to account for parameter uncertainty. Beta distributions were fitted to all probabilities and HRQoL utilities and Gamma distributions were fitted to all cost parameters. A Monte Carlo Simulation with 5,000 iterations was performed. For cost parameters gamma distributions were used and a beta distribution was used for probabilities as well as for health state utilities. Outcomes of the PSA were presented in a cost effectiveness plane.

### Scenario analyses

Model assumptions were changed to determine the health economic impact of the innovation in different use cases or by using different input. For this analysis two scenario analyses were conducted: 1) Exclusion of productivity costs, 2) Calculation of productivity costs with the friction method, as advised by Dutch regulators [16] compared to the approach taken in the main analysis.

## Results

### Base case results

#### Adults

**Fig 2:**
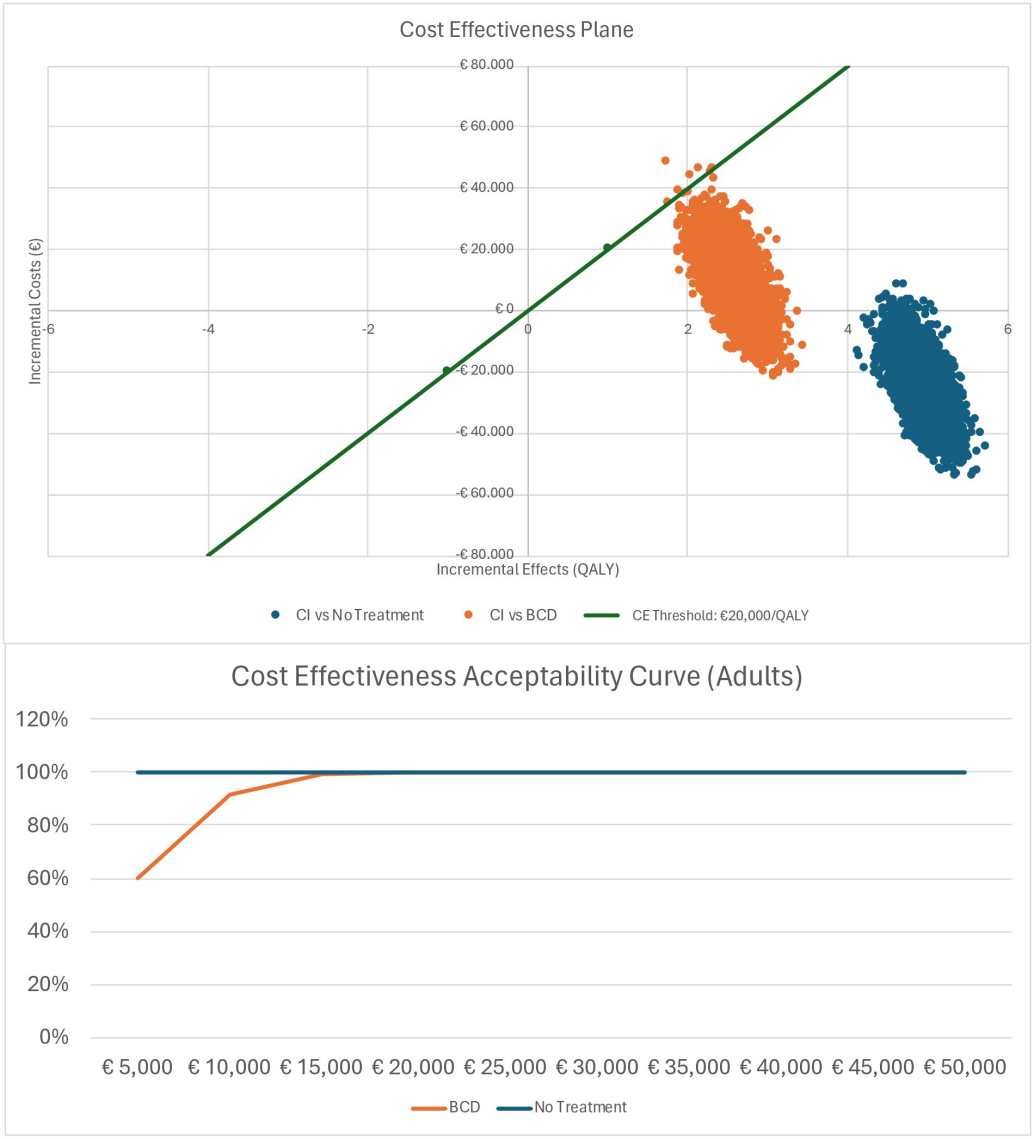
a) Cost-effectiveness plane & b) Cost Effectiveness Acceptability Curve—Adults.

Table 4 shows the discounted results of the base case analysis. For adults with SSD and an average age of 53 years, total costs for CI were €194,051 (95%-CrI €177,274 to €211,108) compared to total BCD costs of €185,310 (95%-CrI €182,367 to €194,142) resulting in additional costs of €8,826 (95%-CrI -€5,020 to €18,252). For no treatment total costs were €219,350 (95%-CrI €216,350 to €226,262) resulting in a difference of -€25,089 (95%-CrI -€31,678 to -€6,003) compared to CI. Over a lifetime, adults with CI gained 18.41 QALYs (95%-CrI 18.07 to 18.75) compared to adults with BCD who gained 15.81 QALYs (95%-CrI 15.53 to 16.10) resulting in 2.60 (95%-CrI 2.15 to 3.05) additional QALYs for CI. Compared to no treatment the difference in QALY was 4.95 (95%-CrI 4.87 to 5.01), as no treatment resulted in 13.46 QALY (95%-CrI 13.20 to 13.73). The ICER for adults receiving CI compared to BCD was €3,494/QALY gained. The ICER remained below the Dutch threshold of €20,000/QALY. No treatment was dominated by CI as costs were lower and health outcomes (QALY) improved. Based on the PSA with 5,000 repetitions a cost-effectiveness plane was constructed, Fig 2a. For BCD 99.9% of model runs resulted in an ICER below the threshold of €20,000/QALY (green line). For no treatment all iterations were well below the threshold. The cost effectiveness acceptability curve shows the probability of an innovation being cost-effective against different thresholds. Fig 2b shows that there is a 100% probability for CI to be cost-effective at a cost-effectiveness threshold of €15,000.

**Table 4:** Base case results—Adults.

	CI		BCD		No Treatment		CI vs BCD	CI vs No Treatment
	Mean	95%-CrI	Mean	95%-CrI	Mean	95%-CrI	Mean	Mean
Direct care	€ 106,621	(€ 92,903–€ 121,637)	€ 25,167	(€ 21,876–€ 28,804)	€ 0	(€ 0–€ 0)	€ 81,454	€ 106,621
Aftercare	€ 5,768	(€ 5,065–€ 6,514)	€ 7,277	(€ 6,253–€ 8,393)	€ 0	(€ 0–€ 0)	-€ 1,509	€ 5,768
Complications	€ 1,271	(€ 1,271–€ 1,510)	€ 245	(€ 218–€ 274)	€ 0	(€ 0–€ 0)	€ 1,026	€ 1,271
Tinnitus	€ 522	(€ 424–€ 629)	€ 543	(€ 441–€ 656)	€ 0	(€ 0–€ 0)	-€ 21	€ 522
Vestibular complications	€ 194	(€ 158–€ 234)	€ 201	(€ 164–€ 242)	€ 0	(€ 0–€ 0)	-€ 7	€ 194
Explantation	€ 65	(€ 49–€ 84)	€ 7	(€ 6–€ 10)	€ 0	(€ 0–€ 0)	€ 58	€ 65
Device failure	€ 3,327	(€ 2,716–€ 4,009)	€ 138	(€ 113–€ 166)	€ 0	(€ 0–€ 0)	€ 3,189	€ 3,327
Total healthcare costs	€ 117,647	(€ 104,163–€ 132,796)	€ 33,514	(€ 30,027–€ 37,300)	€ 0	(€ 0–€ 0)	€ 84,133	€ 117,647
Productivity costs	€ 76,484	(€ 67,179–€ 86,076)	€ 151,789	(€ 143,572–€ 159,827)	€ 219,225	(€ 211,823–€ 226,213)	-€ 75,305	€ 76,484
**Total Costs**	**€ 194,136**	**(€ 177,347–€ 212,394)**	**€ 185,310**	**(€ 182,367–€ 194,142)**	**€ 219,225**	**(€ 211,823–€ 226,213)**	**€ 8,826**	-€ 25,089
**QALY**	**18.41**	**(18.07–18.75)**	**15.81**	**(15.53–16.10)**	**13.46**	**(13.20–13.73)**	**2.60**	**4.95**
**ICER (total costs)**							**€ 3,493**	**CI Dominates**

BCD: Bone Conduction Device, CI: Cochlear Implant, ICER: Incremental Cost Effectiveness Ratio ((Cost Intervention-Cost Control)/(QALY Interventiom-QALY Control)), QALY: Quality Adjusted Life Year

#### Children

Table 5 shows the discounted and undiscounted results of the base case analysis for the pediatric population with an average age of 1.5 years. Total costs with a CI were estimated at €303,626 (95%-CrI €173,507 to €484,006) and for BCD at €333,237 (95%-CrI €300,308 to €429,631) resulting in savings of €29,611 (95%-CrI -€126,800 to €54,375). No treatment resulted in €361,284 (95%-CrI €320,194 to €478,087) in costs, a saving of €57,658 (95%-CrI -€146,687 to €5,919) compared to CI.

**Table 5:** Base case results (discounted)—Children.

	CI		BCD		No Treatment		CI vs BCD	CI vs No Treatment
	Mean	95%-CrI	Mean	95%-CrI	Mean	95%-CrI	Mean	Mean
Direct care	€ 136,949	(€ 118,673–€ 156,245)	€ 26,535	(€ 22,870–€ 30,598)	€ 0	(€ 0–€ 0)	€ 110,414	€ 136,949
Aftercare	€ 14,805	(€ 13,046–€ 16,729)	€ 8,269	(€ 7,049–€ 9,649)	€ 0	(€ 0–€ 0)	€ 6,536	€ 14,805
Complications	€ 16,900	(€ 13,743–€ 20,245)	€ 3,890	(€ 3,419–€ 4,368)	€ 0	(€ 0–€ 0)	€ 13,010	€ 16,900
Tinnitus	€ 0	(€ 0–€ 0)	€ 0	(€ 0–€ 0)	€ 0	(€ 0–€ 0)	€ 0	€ 0
Vestibular complications	€ 59	(€ 48–€ 71)	€ 42	(€ 34–€ 51)	€ 0	(€ 0–€ 0)	€ 17	€ 59
Explantation	€ 104	(€ 77–€ 134)	€ 8	(€ 6–€ 11)	€ 0	(€ 0–€ 0)	€ 96	€ 104
Device failure	€ 7,369	(€ 6,004–€ 8,844)	€ 174	(€ 139–€ 213)	€ 0	(€ 0–€ 0)	€ 7	€ 7
Total healthcare costs	€ 176,382	(€ 157,336–€ 196,174)	€ 38,978	(€ 34,756–€ 43,522)	€ 0	(€ 0–€ 0)	€ 137,404	€ 176,382
Productivity costs	€ 124,698	(€ 7,813–€ 297,608)	€ 294,862	(€ 204,453–€ 393,087)	€ 361,284	(€ 320,913–€ 478,087)	-€ 170,164	-€ 236,586
**Total Costs**	**€ 303,626**	**(€ 173,507–€ 484,006)**	**€ 333,237**	**(€ 300,308–€ 429,631)**	**€ 361,284**	**(€ 320,913–€ 478,087)**	**-€ 29,611**	**-€ 57,658**
**QALY**	**36.20**	**(28.78–41.57)**	**28.98**	**(24.59–33.02)**	**26.03**	**(20.82–31.06)**	**7.22**	**10.17**
**ICER (total costs)**							**CI Dominates**	**CI Dominates**

BCD: Bone Conduction Device, CI: Cochlear Implant, ICER: Incremental Cost Effectiveness Ratio ((Cost Intervention-Cost Control)/(QALY Interventiom-QALY Control)), QALY: Quality Adjusted Life Year

Children with CI gained 36.20 (95%-CrI 28.78 to 41.57) QALYs whereas children with BCD gained 28.98 (95%-CrI 24.59 to 33.02) QALYs. Thus, CI results in 7.22 (95%-CrI 4.19 to 8.55) additional QALYs compared to BCD over a lifetime horizon. When no treatment was provided, the difference was 26.03 (95%-CrI 20.82 to 31.06) given a QALY realization of 10.17 (95%-CrI 7.96 to 10.51) for the no treatment patient. Compared to both BCD and no treatment, CI was cost saving and performed better in terms of health outcomes, thus dominating BCD and no treatment. Based on the PSA with 5,000 repetitions a cost-effectiveness plane was constructed, Fig 2a. When CI was compared to BCD, 89.6% of model runs resulted in an ICER below the threshold of €20,000/QALY (green line). When compared to no treatment, 93.1% of iterations were below the threshold.

**Fig 3:**
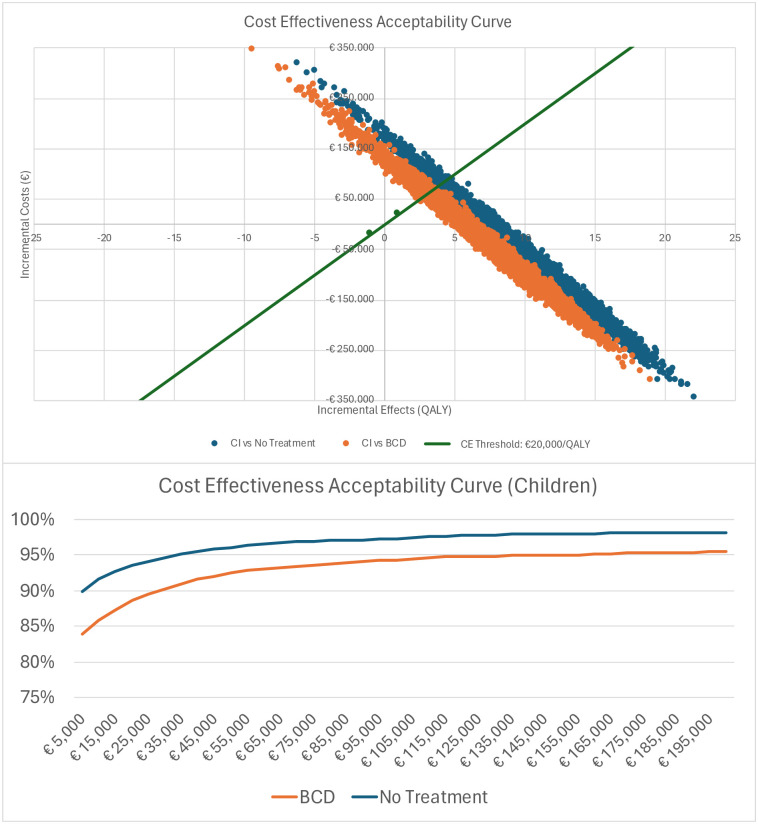
a) Cost-effectiveness plane & b) Cost Effectiveness Acceptability Curve—Children.

The PSA confirmed CI to be the dominant strategy over BCD and no treatment. The CE Acceptability Curve in Fig 3b shows that there is an 89% probability for CI to be cost-effective at a cost-effectiveness threshold of €20,000 when compared to BCD, and a 94% probability when compared to no treatment.

### One-way sensitivity analysis

**Fig 4:**
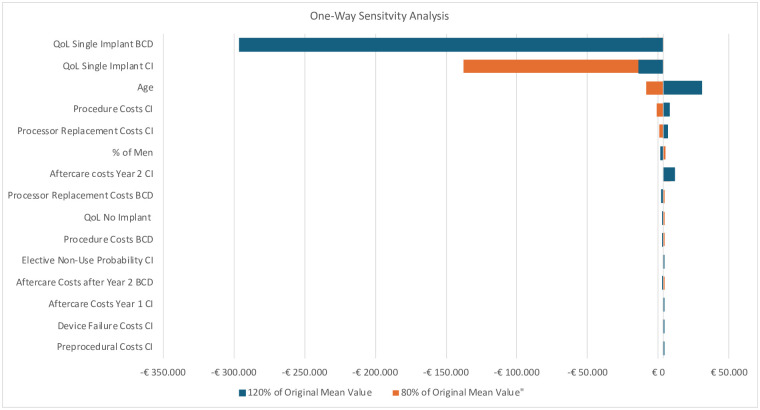
Tornado diagram of the one-way sensitivity analysis (OWSA)—Adults. BCD, Bone Conduction Device, CI: Cochlear Implant, QOL: Quality of Life.

Fig 4 shows the OWSA with the fifteen parameters with the greatest influence on the ICER for adults when CI is compared to BCD. We chose to only present the OWSA for CI vs BCD in adults as the model used in the other comparisons are identical and this sensitivity analysis also shows how sensitive the ICER is to inputs in the other scenarios. The diagram shows that Quality of Life (QoL) utility parameters for BCD and CI had the greatest impact on the ICER due to the difference in QALYs. Besides the QoL parameters and age, all other values remained below the cost-effectiveness threshold of €20,000/QALY.

### Scenario analyses

#### Productivity costs

When productivity costs were excluded from the analysis, the ICERs for the pediatric population remained below the threshold of €20,000 with an ICER of €19,338 for CI vs BCD and €17,481 for CI vs no treatment. In the adult population, when productivity costs were excluded, the ICER was €32,341 for CI vs BCD and €23,767 for CI vs no treatment. When the friction method was applied in the adult population, an ICER of €32,341 for CI vs BCD and €28,202 for CI vs no treatment was found. In the pediatric population the friction method was not applied as it would lead to the same outcomes as excluding productivity costs completely.

#### Age

**Fig 5:**
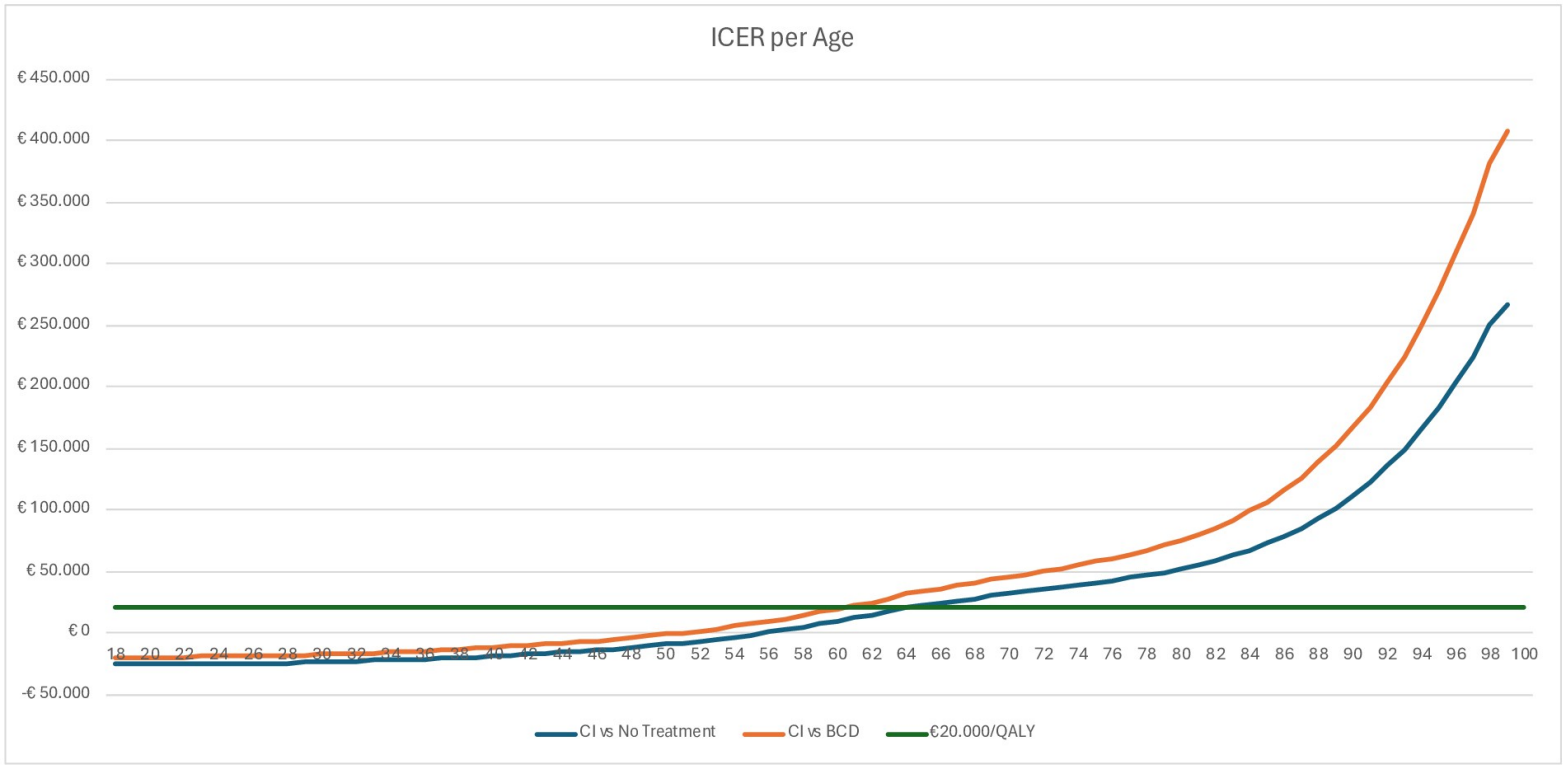
ICER per age for CI vs BCD and CI vs no treatment.

We used the decision analytic models to determine the ICER at different ages. In Fig 5, the age is presented on the X-axis and the ICER on the Y-axis. The ICER is zero (no health benefit and no change in costs) for CI compared to BCD at age 51, and for CI compared to no treatment at age 55. Providing a CI remains a cost-effective strategy, with an ICER below the threshold of €20,000/QALY, until 61 compared to BCD and until 64 when compared to no treatment.

## Discussion

In the cost-effectiveness analysis of CI vs BCD in the adult population CI showed to be cost-effective from a societal perspective with an ICER of €3,493 at the cost-effectiveness threshold of €20,000/QALY. The robustness of the cost-effectiveness results was tested in the probabilistic sensitivity analysis. For the adult population, 99.9% of model runs resulted in an ICER below the threshold indicating high confidence in the conclusions of this Cost Effectiveness Analysis (CEA). For the pediatric population, the probabilistic sensitivity analyses showed a more heterogenous outcome, with 90% of model runs result in an ICER below the threshold. When CI was compared to no treatment, the application of CI in SSD patients was demonstrated to be dominant over no treatment, resulting in both a cost saving as well as improvement of quality of life. This was also confirmed by the PSA, with 100% of iterations resulting in an ICER below the threshold of €20,000/QALY. Again, in the pediatric population the PSA results were more heterogeneous.

From a health system perspective, CI was not cost-effective compared to either BCD or no treatment in adult population, with ICERs above the threshold of €20,000/QALY. For the pediatric population, the ICERs remained below the threshold. The results from the health system perspective compared to the base case analysis show the importance of the productivity costs on outcomes in the base analysis. The total healthcare costs found in our models is comparable to other Dutch CEA for CI [18]. In terms of QALY outcomes our models found larger incremental differences compared to other published studies [11, 25]. This difference was the result of the different utility values used in this analysis, and in the case of the Ontario report due to different time horizons, (lifelong vs 25 years) and differing discount rates. Both our analysis and the Ontario’s analysis showed CI to be cost-effective compared to no treatment, however, the Canadian analysis did not compare CI to BCD in SSD [11].

When the age of patients was varied in our scenario analyses, we found ICERs below the threshold of €20,000/QALY, until 61 when compared to BCD and 64 compared to no treatment. Even though the model, the input parameter values, and the cost effectiveness threshold differed, the ages we found are in line with those found by Dreyfuss et al. [26]. The sensitivity of outcomes to the patients age was confirmed with the OWSA that also showed that for adult patients, age was an influential factor, mainly due to the effects on productivity costs.

In our analysis we used utilities for quality of life based on HUI-3 questionnaires. The use of HUI-3 utilities in this cost-effectiveness analysis is considered a limitation since the Dutch costing guideline recommends the use of Dutch specific EQ-5D utilities [24]. However, since the HUI-3 includes a domain on hearing, it is considered to reflect HRQOL better than the EQ-5D which does not include a hearing specific domain [27]. Utility values for this population in the Netherlands were not available, therefore we used data based on a German population, which may influence model outcomes due to valuation differences. Based on literature it is expected to see less change in HRQOL with the EQ-5D compared to the HUI-3. Therefore, the use of EQ-5D would likely result in less favorable cost effectiveness outcomes for more expensive treatments as the impact on quality of life of CI could be underestimated [27, 28]. Child specific utility values were not available. Therefore, the results of the cost-effectiveness analysis may underestimate the beneficial effect of CI in this younger population as improvements are considered larger in this population due to potential positive effects on developmental outcome [29]. For future research we believe it is beneficial to invest in more research on quality-of-life utilities for the adult and pediatric population.

In our analysis we did not include the potential effect of implanting a CI on the education of children, this may result in an underestimation of the societal effects of CI for SSD. For both the adult and the pediatric population, a human capital approach was chosen to estimate productivity costs to better reflect the long-term impact of impaired hearing on productivity. It is expected that people with a hearing impairment will not be replaced at work after a short period, as calculated by the friction method, but continue to work, however, less optimally (presenteeism) and in lower functions. Furthermore, absenteeism was linearly linked to health-related quality of life utilities, an approach that may lead to over-estimation of the productivity costs. Scenario analyses with use of the friction method showed similar outcomes compared to the health system perspective. More data on educational effects and productivity effects is therefore desirable to reduce uncertainty of the societal effects of CI for SSD.

No country specific clinical outcomes for explantation risk and non-use of the CI and BCD were available but were assumed to be comparable across countries.

## Conclusion

Based on the results of the model, it is very likely that CI is cost-effective compared to BCD in the Dutch adult and pediatric population with SSD at a threshold of €20,000/QALY suggesting CI should be reimbursed by insurance up to an age of 61 for persons with SSD. The sensitivity analyses and scenarios demonstrate the robustness of model outcomes.
